# A Stepwise Proposal for Low-Grade Hemorrhoidal Disease: Injection Sclerotherapy as a First-Line Treatment and Rubber Band Ligation for Persistent Relapses

**DOI:** 10.3389/fsurg.2021.782800

**Published:** 2022-01-10

**Authors:** Roberta Tutino, Marco Massani, Leonel Jospin Kamdem Mambou, Paolina Venturelli, Immacolata Della Valle, Giuseppina Melfa, Matilde Micheli, Gaia Russo, Gregorio Scerrino, Sebastiano Bonventre, Gianfranco Cocorullo

**Affiliations:** ^1^Department of Surgical, Oncological and Stomatological Disciplines, University of Palermo, Palermo, Italy; ^2^Chirurgia 1, Ospedale Regionale di Treviso, Azienda ULSS 2 Marca Trevigiana, Treviso, Italy

**Keywords:** hemorrhoids, hemorrhoid ligation, hemorrhoid sclerotherapy, review, outpatient treatment, hemorrhoid complications

## Abstract

Outpatient treatments are actually the techniques of choice in the management of low-grade hemorrhoidal disease. Among these, rubber band ligation (RBL) and injection sclerotherapy (IS) are the most frequently performed. Both techniques are used, without one having been determined to be superior over the other. We analyzed the studies that compare these two techniques in terms of efficacy and safety in order to offer a proposal for treatment choice. RBL seems to be most efficient in terms of symptom resolution for second-degree hemorrhoidal disease and equal or superior for treatment of third-degree disease. However, IS offers lower rates of severe post-operative pain and minor complications. Since outpatient treatments are offered to patients as painless options that allow a prompt recovery, we propose a stepwise protocol using 3% polidocanol or aluminum potassium sulfate and tannic acid IS as the first treatment option, as it has less complications, followed by RBL in cases of relapse.

## Graphical Abstract

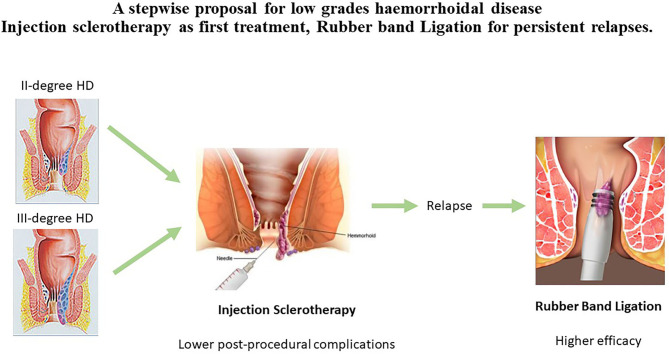

## Introduction

Patients suffering from hemorrhoidal disease (HD) would can obtain rapid and efficient symptom resolution with low rates of post-operative complications and recurrence (1). In order to provide the correct care for these patients, it is necessary to find a balance between the resolution of symptoms and post-operative morbidity, explaining to the patient that recurrence or incomplete resolution of HD can be treated again. Rubber band ligation (RBL) and injection sclerotherapy (IS) are the most commonly used non-surgical techniques for HD. These are recommended by national and international guidelines (2, 3) for the management of low-grade HD (II–III according to Goligher's classification), (4) while no role for these techniques is recognized in the management of complicated hemorrhoids (5).

The treatments can be offered in an office-based modality and are cost and time-saving techniques that allow the preservation of the working days of patients and avoid the post-operative morbidity associated with hemorrhoidectomy. Unfortunately, these treatments are also not entirely free from complications. Based on previous systematic reviews, post-operative pain in RBL ranges from 8 to 80% and post-operative bleeding is reported in up to 50%. For IS, post-procedural pain is reported in 36–46% of patients, while no post-operative bleeding has been described (6, 7). The COVID-19 pandemic, with its restrictions on hospital admissions of patients, has further strengthened the need for appropriate selection of treatment for HD (8).

Rubber band ligation (RBL) and IS are offered to patients according to the personal choice of their surgeon. Thus, there is likely a need to evaluate the available techniques and develop a consistent method for deciding on their use in accordance with a proper classification (9). The aim of this analysis is to offer a proposal for the use of the office-based treatments, evaluating whether IS and RBL are comparable or if one should be preferred among them. Most of the studies involving IS and RBL are retrospective and few are contemporary. However, some new data are now available from recent randomized controlled trials (RCTs). We performed an up-to-date literature review of studies that compare RBL and IS with the aim of developing a standardized protocol for the management of grade II–III HD. This literature review was undertaken in accordance with the Preferred Reporting Items for Systematic Reviews and Meta-Analyses (PRISMA) guidelines (10).

**Eligibility criteria**. Studies on patients complaining of bleeding or prolapse due to HD comparing RBL and IS were collected. The hemorrhoidal degree and the presence of bleeding or prolapse were analyzed. The well-defined treatment modality, post-procedural complications, and the symptom resolution rates as outcome measures were checked in the articles, and the follow-up period was considered. The literature search comprised all original papers published from January 2000 to June 2021. No language selection was implemented. The exclusion criteria were lack of information on hemorrhoidal degree, post-operative morbidity, or recurrence.

**Information source**. Original papers were identified by searching PubMed or MEDLINE database and the Cochrane library database.

**Search strategy**. The search terms used were: Hemorrhoids, Hemorrhoid ligation, Hemorrhoid sclerotherapy, Review, and Outpatient treatment.

### Study Records

**Data management**. An electronic record using an Excel framework was made, including sample sizes and initial numbers.

**Selection process**. Articles were searched by two independent reviewers for initial screening and eligibility before inclusion in the review.

**Data collection process**. Data were extracted using a pilot form and then selected according to the missing or superfluous ones. Disagreements among reviewers were resolved by discussion.

**Data items**. Number of patients, patient demographic data, hemorrhoidal degree according to Goligher's classification, the occurrence of post-treatment complications, and the recurrence rate at follow-up were recorded.

**Outcomes and prioritization**. The occurrence of post-operative complications, such as pain and bleeding and the recurrences at follow-up, were the main measured outcomes.

**Risk of bias in individual studies**. Some reports grouped the hemorrhoidal degrees; this can confound results related to singular degrees. Loss of patients, short periods of follow-up and differences in outcome measures can lead to bias at the outcome level.

## Technical Notes

### RBL

Rubber band ligation (RBL) should be avoided in patients with other anorectal diseases such as fistulas, thrombosed hemorrhoids and fissures, immunodeficient patients, and in those with coagulation disorders. No data on pregnant women are present in the literature (6, 7).

Rubber band ligation (RBL) can be performed by suction or by forceps. Ramzisham compared forceps vs. suction ligations and highlighted how pain during and after the first 24 h was worse with the use of forceps. The forceps procedure was also associated with more intra-procedural bleeding (11). Cazemeier and Wehrmann evaluated the use of endoscopic ligations and concluded that trans-anal ligation with a proctoscope is low-cost and causes little pain in comparison to the endoscopic procedure, and is comparable in terms of recurrences. However, ligation using a flexible endoscope is easier, offers better maneuverability and the possibility of a photographic documentation, and allows the performance of more ligations (12, 13). Notably, data on the proper number of ligations to be inserted are inconclusive (6). The number of sessions required is one in 63–69% of cases and two in 3–30%. The proper interval between two sessions proposed by literature is 4 weeks (6, 14, 15).

### IS

Injection sclerotherapy (IS) should be avoided in patients with thrombosed hemorrhoids; cardiac, hepatic, renal, or hematological diseases; pregnant or nursing mothers; and people with asthma, allergic predisposition, hypercoagulability, thrombophilia, anticoagulant therapy, or inflammatory bowel disease (6, 7).

Several agents are used for injection therapy for HD: 5% phenol in almond oil, 50% dextrose in water, 3% polidocanol, and aluminum potassium sulfate and tannic acid. Akindiose et al. compared 5% phenol in almond oil and 50% dextrose in water and found comparable results in terms of 6-month symptom resolution (92.3% vs. 89.7%) in patients with grade I–II and III HD (16). In the comparison of 5% phenol in almond oil with aluminum potassium sulfate and tannic acid, the former obtained poorer results at 1-year follow up. In their study, Yano et al. analyzed third-degree HD and found that 80% of patients treated with 5% phenol in almond oil experience recurrence, while use of aluminum potassium sulfate and tannic acid resulted in a resolution of both bleeding and prolapse in 75% of patients (17).

Mishra et al. evaluated 3% polidocanol vs. 5% phenol in almond oil and found low rates of pain during defecation, permanent pain, and pruritus but also higher satisfaction rates with the use of 3% polidocanol. Permanent pain was described in 2.8% of the patients in the 3% polidocanol group and in 4.8% of the group treated with 5% phenol in almond oil (18).

A special foam formulation of 3% polidocanol has recently been proposed (19). Polidocanol is a non-ionic surfactant that mainly targets endothelial cells, causing vasospasm. According to the authors, the foam formulation leads to homogeneous distribution of drug microbubbles. They demonstrated its efficacy in the management of second and third-degree HD, with reported success rates of 78.8% at 1-year follow-up. On the other hand, 13.6% of patients suffered post-procedural pain lasting up to 5 days. In contrast with other techniques, the authors injected the suspension into the piles and not into the submucosa at the base of each hemorrhoidal pile above the dentate line (20).

### Symptom Resolution

Bleeding and prolapse are the principal symptoms suffered by patients with HD. Both IS and RBL are intended to cause local inflammation, which leads to reduced blood flow in the hemorrhoids and fibrosis of the area, retracting the prolapse into the anal canal.

Results of both techniques in terms of symptom resolution are reasonably good and maintained over time. However, patients must be informed of the possible need for second sessions and re-treatment in future.

Kanellos et al. performed an RCT analyzing 161 patients suffering from second-degree HD, comparing RBL and IS with 5% phenol solution in almond oil. Additional sessions for persistent symptoms after 4 weeks were required in 33% of the IS group and 52% of the RBL group (*p* = 0.013). At 6–24 months follow-up, 30% of the patients from the IS group and 17% from the RBL group required further treatments (*p* = 0.06). The results were poorly maintained over time, and at 4-year follow-up, bleeding was present in 81.3% of the IS group and 60.5% of the RBL group (*p* = 0.004), whereas spontaneously or manually reducible prolapse was present in 82.6% of the IS group and 60.4 % of the RBL group (*p* = 0.02). Finally, long term symptom resolution was achieved in only 8% of the IS group and 31% of the RBL group (21).

Jehan et al. performed an RCT comparing RBL and IS in 100 patients with second-degree HD and found symptom resolution in 56% of the patients from the IS group and 88% of the RBL group at 4–6 weeks post-treatment Overall, 32% of the patients in the IS group and 12% of those in the RBL group required a second session. The authors report good results for both techniques at 1-year follow-up, with 100% symptom resolution in the RBL group and 88% in the IS group. Additionally, long-term follow-up showed 100% symptom resolution for RBL vs. 92% in the IS group (*p* = 0.041) (22).

Awad et al. published a prospective comparative study of 120 cirrhotic patients analyzing the efficacy of RBL and two types of IS, ethanolamine oleate 5%, and N-butyl cyanoacrylate, concluding that although RBL was associated with higher satisfaction than IS, there were no statistically significant differences in success rate (23).

Gireboinwad et al. conducted an RCT comparing polidocanol 3% IS, RBL, and hemorrhoidectomy. They collected data from 150 patients with 50 in each treatment group. First and second-degree cases were treated with IS, I–II and III with RBL, and III and IV with hemorrhoidectomy, obtaining 88% improvement or resolution of symptoms after IS and 88% after RBL. However, the data from this study are biased due to the difference in how the groups selected the treated patients (24).

Nasir et al. conducted an RCT comparing IS with 5% phenol in almond oil and RBL in 116 patients with second-degree HD. They describe only a short-term follow-up of 15 days, reporting that 82.1% of the RBL group and 61.3% of the IS group showed symptom resolution. Overall, 54.8% of patients in the IS group and 3.4% of the RBL group required a second treatment (*p* < 0.05) (25).

Abiodun et al. conducted a prospective comparative study analyzing 60 patients with second and third-degree HD, comparing RBL and IS with 50% dextrose in water. They report that anal prolapse was more frequently partially or completely resolved in the RBL group compared to the IS group (*p* = 0.03), while bleeding resolution was more frequent in the IS group (*p* = 0.07). At 3 months, seven patients (23.3%) in the IS group and four (13.3%) in the RBL group required a further treatment (*p* = 0.34) (26).

Makanjoula et al. performed a prospective comparative study on 74 patients with grade I–III HD, comparing IS with 3% polidocanol and RBL at 3-month follow-up. The authors highlight that the two techniques are equally effective for the treatment of patients with grade I–III HD (27). The data are summarized in Table 1.

**Table 1:** Data on symptoms' resolution and need for re-treatment offered by RCTs comparing IS vs. RBL.

**Authors**	**Year**	**Degree**	**N. of patients**	**Type of sclerosant**	**Symptoms' resolution**		**Re-treatment**	
					**IS (%)**	**RBL (%)**	**IS (%)**	**RBL (%)**
Kanellos	2003	II	161	5% phenol in almond oil	67	48	33	52
Jehan	2012	I–II	100	ND	56	88	32	12
Awad	2012	II–III	120	ethanolamine oleate 5%/N-butyl cyanoacrylate	87	90	20	20
Gireboinwad	2014	I–II (IS)/I–III(RBL)	100	polidocanol 3%	88	88	ND	ND
Nasir	2017	II	116	5% phenol in almond	61	82	54	34
Abiodun	2021	II–III	60	50% dextrose in water	46	76.6	23	13
Makanjoula	2021	I–III	74	3% Polidocanol	*P =* 0.391		ND	ND

### Post-procedural Complications

The most frequent procedural and post-procedural complications encountered in both techniques are mild to moderate pain, tenesmus, and bleeding. These complications usually do not impair the ability of the patient to return to work the same day. However, low but not negligible rates of severe anal pain have been reported in the literature, alongside some sporadic cases of life-threatening complications (28).

Kanellos et al. reported an up to 69.1% rate of post-operative minor complications in patients treated with RBL, with a rate of 30% in the IS group (*p* < 0.001). It is notable that severe anal pain was suffered by 11.1% of the RBL group vs. 1.3% of the IS group (21).

Gireboinwad et al. conducted an RCT comparing IS, RBL, and hemorrhoidectomy. Mild post-operative anal pain was present in 12% of RBL patients vs. 4% of IS patients, and early complications were seen in 68% of the RBL group vs. 32% of the IS group (24). In the report by Nasir, moderate pain was more common in the RBL group (5.2 vs. 1.7%) (p > 0.05), while severe pain was equally reported (1.7%) (25).

Abiodun et al. reported that severe anal pain was more frequent and had a longer time to complete resolution in the RBL group. However, this difference was not statistically significant (*p* = 0.35) (26).

In the study by Makanjoula et al., rates of minor complications were similar in both groups (5.7% in RBL vs. 8.1% in IS; *p* = 0.643). The median pain score was significantly higher in the RBL group compared to the IS group after the first and second sessions (*p* < 0.001) (27). Cirrhotic patients undergoing IS showed higher rates of minor complications and severe anal pain only in the study performed by Awad et al. (23). Data are summarized in Table 2.

**Table 2:** Minor and severe pain after IS and RBL procedures.

**Author, year**	**Degree**	**IS**	**RBL**	**Minor complications**		**Severe pain**	
				**IS**	**RBL**	**IS**	**RBL**
Kanellos et al. (21)	II	80	81	30%	69.1%	1.3%	11.1%
Jehan et al. (22)	I-II	50	50	32% (16)	40% (20)	0	0
Awad et al. (23)		60	60	ND	ND	61.6%	20%
Gireboinwad et al. (24)	I-II (IS)/I-III(RBL)	50	50	32%	68%	4%	12%
Nasir et al. (25)	II	58	58	8.6%	10.2%	1.7%	1.7%
Abiodun et al. (26)	II-III	30	30	33.3%	30%	0	13.3%
Makanjoula et al. (27)	I-III	37	37	8.1%	5.7%		>>>

## Discussion

The justification for offering an outpatient treatment for low grade HD is the avoidance of both post-operative complications and the costs of surgical management (i.e., traditional hemorrhoidectomy and its minimally invasive counterpart, arterial ligation).

In terms of efficacy compared to Milligan-Morgan hemorrhoidectomy, non-surgical treatments are equally effective for second-degree HD symptom resolution, but inferior in terms of prolapse resolution for third-degree cases. On the other hand, post-operative pain is present in almost all patients treated by hemorrhoidectomy, with symptoms usually lasting 2 weeks, but in some cases up to 3 months (29).

In comparison to arterial ligation, non-surgical treatments are best for low grades of HD, as stated by the Hubble trial, a UK National Health Service research study that compared hemorrhoidal artery ligation vs. rubber band ligation in terms of cost-effectiveness and serious adverse events (30, 31).

Injection sclerotherapy (IS) and RBL have been considered as two alternative options for the management of low-grade HD, but few data are available to suggest the use of one technique over the other. This is the first review that focuses only on studies that directly compare IS and RBL, offering an evaluation of safety and efficacy.

As summarized in Table 1, we observed that RBL has higher rates of bleeding and prolapse resolution, even though several sclerosant solutions are proposed by the different studies with variable efficacy.

In the analysis of the several sclerosants available, polidocanol 3%, and aluminum potassium sulfate and tannic acid seem to be the most effective (16–20).

The evaluation of efficacy for each hemorrhoidal degree is difficult because most previous studies analyze the results of these techniques in patients with grouped second and third-degree HD. The recent studies by Abiodun and Makanjoula analyzed bleeding and prolapse resolution separately. The first study showed a preference for RBL for prolapse resolution and in IS for bleeding resolution, whereas the second showed a comparable efficacy for both techniques (26, 27).

The present study offers new data that differs from those previously reported by reviews, with both techniques being comparable in term of symptom resolution in second-degree HD, while IS resulted in better outcomes in third-degree cases (7).

Unfortunately, outpatient treatments can cause post-procedural complications. A previous review showed that post-operative complications occurred in 1–50% of RBL cases in the literature, pain in 8–80%, and severe pain in 4–20% (6). In line with these results, our analysis also showed that post-procedural complications and anal pain are more frequent in the RBL groups.

In the overall assessment of IS vs. RBL, no definitive conclusions on the preferable technique can be made, as we have to consider the balance between efficacy and post-operative complications.

Patients must be informed of the potential need for repeated sessions or retreatment, since this is more frequent in patients treated by IS than by RBL, as shown by the results of Abiodun, Nasir, and Jehan (respectively, 23% vs. 13%, 54% vs. 34%, and 32% vs. 12%) (22, 25, 26).

As we must guarantee, to the best of our ability, a prompt recovery of our patients, we propose a stepwise treatment modality when treating patients with low-grade HD. According to our analysis, IS should probably be offered as the first-line treatment option, while RBL should be used in cases of persistent symptomatic second or third-degree anal prolapse.

Since both procedures are not free from relapses and complications, extensive information on efficacy and possible post-operative courses must be given and written consent must be obtained. There is a need for a multicentric RCT to standardize and evaluate the results of these techniques, including separate analysis for second and third-degree HD.

## Author Contributions

RT gave substantial contribution to the conception of the work. RT, LJKM, ID, PV, MM, and GR gave substantial contribution to the acquisition, analysis and interpretation of data. RT, MM, GM, GS, SB, and GC revised critically the work for important intellectual content. All authors gave their final approval of the version to be published and are co-authors of the present paper.

## Conflict of Interest

The authors declare that the research was conducted in the absence of any commercial or financial relationships that could be construed as a potential conflict of interest.

## Publisher's Note

All claims expressed in this article are solely those of the authors and do not necessarily represent those of their affiliated organizations, or those of the publisher, the editors and the reviewers. Any product that may be evaluated in this article, or claim that may be made by its manufacturer, is not guaranteed or endorsed by the publisher.
